# Supplementary material for: Psychological distress, resettlement stress, and lower school engagement among Arabic-speaking refugee parents in Sydney, Australia: A cross-sectional cohort study
Source: PLoS Med. 2021 Jul 12;18(7):e1003512. doi: 10.1371/journal.pmed.1003512 (PMC8312975; doi:10.1371/journal.pmed.1003512)
Supplement: S1 Prospective Protocol — (DOCX) [file pmed.1003512.s001.docx]

**S1: Prospective protocol based on approved ethics submission (HC15833).**

**RECRUITMENT**

Recruitment will be rolling, i.e. across a few school years (See timeline).

1A. REFUGEE SCHOOLCHILDREN AND A PARENT (Parts One and Two)

1B. INCLUSION/EXCLUSION:

To be eligible to participate the children must:

1) Be in Year 5 or 6 at the school (typically 11-12 years old);

2) Have attended the school for between three months to 24 months.

3) Obtained asylum seeker or refugee status in Australia in the last 6-24 months. (If the child’s visa status changes within those three years, for example to resident, the child is still eligible to participate).

4) Speak Arabic or English.

5) Siblings will not be eligible to participate

Parents are eligible to participate if they:

1) have a child who is eligible and keen to participate,

2) Speak Arabic or English

Children and parents who participated in Part One are eligible to participate in Part Two, i.e. participation in Part one does not preclude participants from partaking in Part Two.

1C. STUDY INVITATION

Participating schools will circulate a recruitment advert in their relevant newsletters/ communications inviting parents to contact the school should they wish to participate or find out more information. People with refugee backgrounds do not typically respond to recruitment ads, thus school staff (likely the bilingual Community Liaison Officers, or EADL Teachers) will also approach eligible families to tell them about the study. The NSW Department of Education and all participating schools are happy with this recruitment approach and agree that it is the best strategy (see Letters of Support). Staff will emphasise that participation is voluntary, and that declining to participate will not affect their relationship with the school or UNSW.

2. CONSENT

If parents are keen for their child to participate, school staff (likely the bilingual Community Liaison Officers or EADL Teachers) will invite them to contact the research team and/or to provide verbal assent for a research team member to contact them. School staff can also explain the study to them and arrange for a time to meet with a research team member (and interpreter if applicable). A research team member and/or interpreter will go through the Information and Consent form in the participants language of choice, and will probe potential participants’ understanding of the study, their appreciation of the consequences of participation, and their ability to consider alternative choices and to make a reasoned choice, before obtaining written parental consent for themselves and for their child.

There are separate tick boxes in the Information and Consent form (see attachments) to highlight that: 1) interviews will be recorded (Part One); 2) they will be contacted again in approximately 6 months’ time (Part Two); and 3) a teacher who knows the student well will be invited to report on the participating child. It will be possible for the parent/child to consent to participating in the study, but not to consent to information about the child being collected from a teacher, i.e. for that child, only parent and child report will be obtained, no teacher report will be obtained. We have combined parental consent for the child and own consent (i.e. the parent/guardian's own participation) into the one form. We felt that this would be easier and less overwhelming for the participant, rather than having several pages of repetitive material to read.

Children will give verbal assent at the start of the research session. For the interviews (Part One), the interviewer will play back a short recording of them speaking, as part of a fun icebreaker, but also to confirm that children (and parents) understand that they are being audio-recorded. If the parent gives consent, but then the child decides that he/she does not wish to participate, then we will not proceed with the research session. Similarly, if a child (or parent) decides partway through the research session, that they do not wish to participate, we shall end the session. We will reassure the child and parent that this is fine, validate any concerns that they have, and thank them for their participation thus far.

3. SCREENING - N/A

-------------------------------------------------------------------------------------------------------

1A. PRIMARY SCHOOL STAFF PARTICIPANTS (Parts One and Two)

1B. INCLUSION/EXCLUSION

All school staff speak English.

- Staff who are a Year 6 Teacher, EALD Teacher, Community Liaison Officer, School Counsellor, and who work directly with refugee children at the school are eligible to participate in the focus groups (Part One).

- A Year 6 Teacher or EALD teacher who self-identifies and/or is identified as the Principal as knowing the participating child well, are eligible to participate in the questionnaires (Part Two).

- The Deputy Principal or Principal will be the only staff member specifically eligible to complete the Refugee Readiness Audit with the CI (Part Two).

1C. STUDY INVITATION

Eligible school staff will be invited to participate via two options:

1) By way of an informal information session presented by the CI, where school staff will have the opportunity to ask any questions and take an Information and Consent form away with them.

2) The Deputy Principal or Principal will also approach eligible staff to tell them about the study.

All the schools are happy with this recruitment approach and agree that it is the best strategy (see Letters of Support). It will be emphasised to staff that participation is voluntary, and that declining to participate will not affect their relationship with the school or UNSW.

2. CONSENT

If staff are keen to participate, the CI or the (Deputy) Principal will invite them to contact the research team and/or to provide verbal assent for a research team member to contact them. A research team member will go through the Information and Consent form and probe potential participants’ understanding of the study, their appreciation of the consequences of participation, and their ability to consider alternative choices and to make a reasoned choice, before obtaining their written consent.

3. SCREENING - N/A

----------------------------------------------------------------------------------------------------------

1A. SECONDARY SCHOOL STAFF PARTICIPATION (Part Two: Time 2)

1B: INCLUSION/EXCLUSION:

All school staff speak English.

- A Year 7 Teacher who teaches at the secondary school that the participating child attends, who self-identifies and/or is identified as the Principal as knowing the participating child well, are eligible to participate in the questionnaires.

- The Deputy Principal or Principal of the secondary school that the participating child attends, will be the only staff member specifically eligible to complete the Refugee Readiness Audit with the CI.

1C: STUDY INVITATION

i) SCHOOL

With verbal assent reaffirmed by the participating parent and child at Time 2 (written consent will be obtained at Time 1), the CI will invite the Deputy Principal or Principal of the Secondary school that the child is attending, to participate in the study. (Note: if verbal assent is not obtained from the parent and child at Time 2, this step will not occur and no school/teacher report will be obtained). If agreeable, the Deputy Principal/Principal will be asked to provide a written Letter of Support for the project, which shall be forwarded onto the appropriate ethics committees. It is anticipated that most participating students will move onto the same Secondary School within the catchment area. The NSW Department of Education are happy with this approach, and also offered to assist us in approaching schools or providing school contact details.

ii) SCHOOL STAFF

As above, eligible school staff will be invited to participate via two options:

1) By way of an informal information session presented by the CI, where school staff will have the opportunity to ask any questions and take away an Information and Consent form.

2) The Deputy Principal or Principal will also approach eligible staff to tell them about the study.

It will be emphasised to staff that participation is voluntary, and that declining to participate will not affect their relationship with the school or UNSW.

2. CONSENT

If staff are keen to participate, the CI or the (Deputy) Principal will invite them to contact the research team and/or to provide verbal assent for a research team member to contact them. A research team member will go through the Information and Consent form and probe potential participants’ understanding of the study, their appreciation of the consequences of participation, and their ability to consider alternative choices and to make a reasoned choice, before obtaining their written consent.

3. SCREENING - N/A

**REIMBURSEMENT**

Parent and child participants (Part One and Two):

Each parent-child dyad will receive a $30 Big W gift voucher, to thank them for their participation.

School Staff participants:

Part One: The staff will be provided with food and drinks during the focus groups.

Part Two: The school will receive a $50 gift voucher, as a thank you to the staff for their participation.

**PROJECT DESCRIPTION**

Design: Clustered cohort longitudinal within-subjects design.

Participants: Parents and Children from refugee backgrounds: 400 children and one of their parents from a refugee background will be recruited. Recruitment will be rolling over a few school years. Eligibility criteria and recruitment/consent strategy are described in detail in section 2.7.

School Staff: One Year 6 (Time 1) or Year 7 (Time 2) Teacher or EADL teacher who self-identifies and/or is identified by the Principal as knowing the participating child well will be invited to participate in teacher reports about the child. The Deputy Principal or Principal will be invited to complete the Refugee Readiness Audit with the CI. Eligibility criteria and recruitment/consent strategy are described in detail in section 2.7. a

Measures (see Table of Measures in Appendix): A preliminary selection of measures is presented in the Table of Measures (see attached). They are preliminary in that they may be revised after the findings from Part 1. Specifically, we want to make sure that the measures capture what is meaningful from the perspective of refugee families and school staff. We politely ask the ethics committee to review the current application (and selection of measures) as is, and we confirm that any change to measures or inclusion of additional measures will be submitted as a modification and will not be used until ethics approval obtained.

Existing validated translated versions of parent/child questionnaires will be used where available (see footnote to table). All other parent/child questionnaires will be translated by accredited persons and workshopped in a study group of bilingual team members to confirm meaningful and culturally sensitive translation. The questionnaires will be delivered in an interview format, i.e. a bilingual team member will ask the questionnaire items in the participants’ choice of language and record their answers. The one exception is the trauma questionnaires. These will be delivered as a self-report for parent/child to complete, as our extensive experience in this field suggests that participants find this to be the most agreeable and sensitive way to report trauma history. Teachers will complete the questionnaires, as questionnaires, in English.

Procedure: Child and parent will complete the measures twice; once in Terms 3/4 of Year 5 or 6, and once again in Term 2 of Year 7 (see timeline attached). Parent/child contact details will be obtained at Time 1 with the understanding that the research team will contact them again in approximately 6 months’ time to invite them to participate in a second interview. A separate tick box on the Information and Consent form will confirm that parents understand that they will be contacted again in 6 months’ time. A separate tick box on the Information and Consent form will also ask parents to consent to a teacher who knows the student well to complete a questionnaire about the participating child. Only with this consent (i.e. if no consent to this aspect of the study, a teacher will not be approached – see Section 2.7 for more details), will a Year 6 Teacher complete their teacher report on the child at Time 1, and a Year 7 Teacher complete their teacher report on the child at Time 2. In Year 5 or 6 (Time 1) the parent/child interviews will take place at school, after-hours or during lunchtime. In Year 7 (Time 2), parent/child participants will be offered the choice to be interviewed at the school, a local library or Liverpool Hospital. The child and their parent/guardian will be interviewed separately and brought together at the end. The parent- child dyad will receive a $30 gift voucher. The school will receive a $50 gift voucher.

Participant recruitment will be rolling; if we do not recruit a sufficient number of Year 5 or 6 students in 2016, we shall continue recruitment following the same procedure into 2017 (and then 2018 for the Year 7, i.e. Time 2, follow-up).

--------------------------------------------

5. DATA ANALYSIS PLAN

Data will be analysed using SPSS and Mplus software. Descriptive analyses and correlations will be undertaken. Multi-level modelling will look at school climate predictors of child/parent emotional health/biacculturation over time. Dyadic analyses (e.g. Actor-Partner Interpendence Model) will examine the relationship between parent and child emotional health/biacculturation.
