# Supplementary material for: Psychological distress, resettlement stress, and lower school engagement among Arabic-speaking refugee parents in Sydney, Australia: A cross-sectional cohort study
Source: PLoS Med. 2021 Jul 12;18(7):e1003512. doi: 10.1371/journal.pmed.1003512 (PMC8312975; doi:10.1371/journal.pmed.1003512)
Supplement: S1 Data Tables — (DOCX) [file pmed.1003512.s003.docx]

**S1 Data Tables**

**Table A.** Starting Set of Items Pertaining to Parent School Engagement, the Cultural Broker-Parent Relationship, and Parent Sense of School Belonging.

| **Refugee Parent School Engagement Items** | **Cultural Broker-Parent Relationship Items** | **Refugee Parents’ Sense of School Belonging Items** |
| --- | --- | --- |
| ***School Communication*** |  | 4.1 I feel like a part of the school community |
| 5.1.1 The interpreters available through the school are good/effective | 5.2.1 XX has been important in making me feel secure in the school because xx understands my family's religious and cultural backgrounds | 4.2 People notice that I have something to contribute to the school community |
| 5.1.3 It would be difficult for me to find a way to discuss an issue with school staff (R) | 5.2.2 When we first enrolled at this school, I felt comfortable because xx was there to assist us | 4.3 It is hard for people like me to be accepted by school community |
| 5.1.4 Despite the language restrictions I feel adequately informed about what happens at the school | 5.2.3 I can contact xx if I need to | 4.4 Staff at the school take my opinions seriously |
| 5.1.5 I feel that language restricts how much I can know about my child's progress (R) | 5.2.4 XX helps me with my family's wellbeing and issues not related to school | 4.5 Most teachers at school are interested in me |
| 5.1.6 I cannot be involved with the school as much as I would like because there are not enough non-English speaking staff | 5.2.5 I only feel comfortable meeting with other staff, like the principal or classroom teacher, if xx is there | 4.6 Sometimes I feel as if I don't belong within the school community |
| ***School Assistance*** | 5.2.6 If it weren't for xx I would not know how to engage with the school | 4.7 There is at least one teacher or staff member I can talk to at school if I have a problem |
| 5.3.1 School staff direct me to services I need or that might be helpful to access |  | 4.8 People at school are friendly to me |
| 5.3.2 School staff assist me to read or fill out other non-school related forms, for e.g., Centrelink forms or housing applications |  | 4.9Teachers here are not interested in people like me |
| 5.3.3 The school helped or helps with the cost of uniforms or sports kit |  | 4.10 I am included in lots of activities at ___ school. |
| 5.3.4 The school sometimes provides my child with a free lunch |  | 4.10 I am included in lots of activities at ___ school. |
| 5.3.5 My child misses opportunities in the school because we cannot afford all the costs |  | 4.11 I am treated with as much respect as other parents in the school |
| ***Parent Engagement*** |  | 4.12 I feel very different from most other people at school |
| 5.4.1 I have volunteered at the school (negatively correlated with many items) |  | 4.13 I can really be myself at school |
| 5.4.2 I regularly attend school events |  | 4.14 Teachers at school respect me |
| 5.4.3 I have attended a multicultural day or event at the school, for e.g., Harmony Day |  | 4.15 I wish that my family and I were involved in/ attended a different school in Australia |
| 5.4.4 I have attended parent-teacher interviews |  | 4.16 I feel proud to be a part of the school community |
| 5.4.5 The school is good at involving parents in activities |  |  |
| 5.4.6 The school could do more to make me feel welcome and encouraged to participate |  |  |
| ***Facility Social Capital*** |  |  |
| 5.5.1 The school has provided opportunities to meet and make friends with established Australians |  |  |
| 5.5.2 Through the school, I have met and made friends with people from similar backgrounds to me |  |  |
| 5.5.3 When I visit the school, I am likely to run into friends and acquaintances |  |  |
| 5.5.4 If I need advice about the school, I know other parents who I could call |  |  |
| ***Acculturation*** |  |  |
| 5.6.1 This school helps me feel like I belong in Australia |  |  |
| 5.6.2 Through the school, I have improved my English language skills |  |  |
| 5.6.3 Because of activities through the school (such as excursions) I better understand Australian customs |  |  |

**Table B.** Correlation matrix among Refugee Parent School Engagement Items based on Confirmatory Factor Analysis.

|  | **Scales and Correlations** | | | | |
| --- | --- | --- | --- | --- | --- |
| **Refugee Parent School Engagement Items** | **Communication** | **Assistance** | **Parent Engagement** | **Facilitating Social Capital** | **Acculturation** |
| School Communication (5 items) | 1.0 |  |  |  |  |
| School Assistance (2 items) | 0.96** | 1.0 |  |  |  |
| Parent Engagement (4 items) | 0.25** | 0.29* | 1.0 |  |  |
| School as Facilitating Social Capital (4 items) | 0.22* | 0.47** | 0.34** | 1.0 |  |
| Acculturation (3 items) | 0.10 | 0.19 | 0.31** | 0.68** | 1.0 |
| *Correlation co-efficient is significant at p<0.05; **p<0.01. | | | | | |

**Table C.** School Internal Engagement Scale-Refugee Parent (SIES-RP) [Communication, Assistance, Engagement]: list of all original items and final items retained (including reasons for exclusion of items) through CFA (11 out of 16 items retained for final).

| **School Internal Engagement Scale-Refugee Parent (SIES-RP) scale items** | Model 1  (16 items) | Model 2  (11 items) |  |
| --- | --- | --- | --- |
|  | Estimates | Estimates | Reasons for exclusion of items |
| **School Communication (5 items)** |  |  |  |
| 5.1.1 The interpreters available through the school are good/effective | 0.37** | 0.37** |  |
| 5.1.3 It would be difficult for me to find a way to discuss an issue with school staff (R) | 0.50** | 0.48** |  |
| 5.1.4 Despite the language restrictions I feel adequately informed about what happens at the school | 0.58** | 0.60** |  |
| 5.1.5 I feel that language restricts how much I can know about my child's progress (R) | 0.44** | 0.45** |  |
| 5.1.6 I cannot be involved with the school as much as I would like because there are not enough non-English speaking staff | 0.61** | 0.60** |  |
| **School Assistance (5 items)** |  |  |  |
| 5.3.1 School staff direct me to services I need or that might be helpful to access | 0.48** | 0.49** |  |
| 5.3.2 School staff assist me to read or fill out other non-school related forms, for e.g. Centrelink forms or housing applications | 0.10 | Excluded | Insignificant in Model 1 and excluded |
| 5.3.3 The school helped or helps with the cost of uniforms or sports kit | 0.29** | 0.27** |  |
| 5.3.4 The school sometimes provides my child with a free lunch | 0.02 | Excluded | Insignificant in Model 1 and excluded |
| 5.3.5 My child misses opportunities in the school because we cannot afford all the costs | 0.12 | Excluded | Insignificant in Model 1 and excluded |
| **Parent Engagement (6 items)** |  |  |  |
| 5.4.1 I have volunteered at the school (negatively correlated with many items) Excluded – not significant | 0.03 | Excluded | Insignificant in Model 1 and excluded |
| 5.4.2 I regularly attend school events | 0.27** | 0.27** |  |
| 5.4.3 I have attended a multicultural day or event at the school, for e.g. Harmony Day | 0.22** | 0.23** |  |
| 5.4.4 I have attended parent-teacher interviews | 0.18* | 0.19* |  |
| 5.4.5 The school is good at involving parents in activities | 0.13 | Excluded | Insignificant in Model 1 and excluded |
| 5.4.6 The school could do more to make me feel welcome and encouraged to participate | 0.50* | 0.50** |  |
| **Model summary** |  |  |  |
| Comparative Fit Index CFI) | 0.47 | 0.60 |  |
| Tucker-Lewis Index (TLI) | 0.38 | 0.49 |  |
| Standardized Root Mean Square Residual (SRMR) | 0.09 | 0.08 |  |
| Akaike Information Criteria (AIC) | 9971.0 | 6834.6 |  |
| Bayesian (BIC) Adjusted for sample size | 9984.1 | 6843.6 |  |
| Note: (*) and (**) indicate estimates statistically significant at p<0.05 and p<0.01. | | | |

**Table D.** School Community Engagement Scale – Refugee Parent (SCES-RP) [Facility social capital and Acculturation]: list of all original items and final items retained (including reasons for exclusion of items) through CFA (all 7 items retained for final).

| School Community Engagement Scale – Refugee Parent (SCES-RP) scale items | Model 1 (7 items) | Reasons for exclusion of items |
| --- | --- | --- |
|  | Estimates |  |
| **Facility Social Capital (4 items)** |  |  |
| 5.5.1 The school has provided opportunities to meet and make friends with established Australians | 0.64** |  |
| 5.5.2 Through the school, I have met and made friends with people from similar backgrounds to me | 0.74** |  |
| 5.5.3 When I visit the school, I am likely to run into friends and acquaintances | 0.44** |  |
| 5.5.4 If I need advice about the school, I know other parents who I could call | 0.50** |  |
| **Acculturation (3 items)** |  |  |
| 5.6.1 This school helps me feel like I belong in Australia | 0.25* |  |
| 5.6.2 Through the school, I have improved my English language skills | 0.59** |  |
| 5.6.3 Because of activities through the school (such as excursions) I better understand Australian customs | 0.50** |  |
| **Model summary** |  |  |
| Comparative Fit Index CFI) | 0.84 |  |
| Tucker-Lewis Index (TLI) | 0.75 |  |
| Standardized Root Mean Square Residual (SRMR) | 0.06 |  |
| Akaike Information Criteria (AIC) | 4519.9 |  |
| Bayesian (BIC) Adjusted for sample size | 4525.6 |  |
| Note: (*) and (**) indicate estimates statistically significant at p<0.05 and p<0.01. | | |

**Table E.** Cultural Broker Relationship Scale (CBRS): list of all original items and final items retained (including reasons for exclusion of items) through CFA (5 out of 6 items retained).

| Cultural Broker Relationship Scale (CBRS) items | Model 1  (6 items) | Model 2  (5 items) | Reasons for exclusion of items |
| --- | --- | --- | --- |
|  | Estimates | Estimates |  |
| 5.2.1 xx has been important in making me feel secure in the school because xx understands my family's religious and cultural backgrounds | 0.73** | 0.73** |  |
| 5.2.2 When we first enrolled at this school, I felt comfortable because xx was there to assist us | 0.80** | 0.80** |  |
| 5.2.3 I can contact xx if I need to | 0.43** | 0.42** |  |
| 5.2.4 XX helps me with my family's wellbeing and issues not related to school | 0.13 | Excluded | Insignificant in Model 1 and Excluded |
| 5.2.5 I only feel comfortable meeting with other staff, like the principal or classroom teacher, if xx is there | 0.45** | 0.46** |  |
| 5.2.6 If it weren't for xx I would not know how to engage with the school | 0.33** | 0.34** |  |
| **Model summary** |  |  |  |
| Comparative Fit Index CFI) | 0.79 | 0.81 |  |
| Tucker-Lewis Index (TLI) | 0.65 | 0.62 |  |
| Standardized Root Mean Square Residual (SRMR) | 0.08 | 0.08 |  |
| Akaike Information Criteria (AIC) | 2820.5 | 2166.1 |  |
| Bayesian (BIC) Adjusted for sample size | 2824.3 | 2169.2 |  |
| Note: (*) and (**) indicate estimates statistically significant at p<0.05 and p<0.01. | | | |

**Table F.** School Belonging Scale – Refugee Parent (SBS-RP): list of all original items and final items retained (including reasons for exclusion of items) through CFA (15 out of 16 items retained).

| School Belonging Scale – Refugee Parent (SBS-RP) items | Model 1  (16 items) | Model 2  (15 items) | Reasons for exclusion of items |
| --- | --- | --- | --- |
|  | Estimates | Estimates |  |
| 4.1 I feel like a part of the school community | 0.64* | 0.64* |  |
| 4.2 People notice that I have something to contribute to the school community | 0.03 | Excluded | Insignificant in Model 1 and Excluded |
| 4.3 It is hard for people like me to be accepted by school community | 0.33* | 0.33* |  |
| 4.4 Staff at the school take my opinions seriously | 0.44** | 0.44** |  |
| 4.5 Most teachers at school are interested in me | 0.61** | 0.61** |  |
| 4.6 Sometimes I feel as if I don't belong within the school community | 0.57** | 0.57** |  |
| 4.7 There is at least one teacher or staff member I can talk to at school if I have a problem | 0.48** | 0.48** |  |
| 4.8 People at school are friendly to me | 0.62** | 0.62** |  |
| 4.9Teachers here are not interested in people like me | 0.47** | 0.47** |  |
| 4.10 I am included in lots of activities at ___ school. | 0.15* | 0.15* |  |
| 4.11 I am treated with as much respect as other parents in the school | 0.54** | 0.54** |  |
| 4.12 I feel very different from most other people at school | 0.39** | 0.39** |  |
| 4.13 I can really be myself at school | 0.42** | 0.42** |  |
| 4.14 Teachers at school respect me | 0.59** | 0.59** |  |
| 4.15 I wish that my family and I were involved in/ attended a different school in Australia | 0.57** | 0.57** |  |
| 4.16 I feel proud to be a part of the school community | 0.69** | 0.70** |  |
| **Model summary** |  |  |  |
| Comparative Fit Index CFI) | 0.74 | 0.76 |  |
| Tucker-Lewis Index (TLI) | 0.7 | 0.72 |  |
| Standardized Root Mean Square Residual (SRMR) | 0.073 | 0.072 |  |
| Akaike Information Criteria (AIC) | 8185.7 | 7435.3 |  |
| Bayesian (BIC) Adjusted for sample size | 8199.0 | 7447.7 |  |
| Note: (*) and (**) indicate estimates statistically significant at p<0.05 and p<0.01. | | | |

**Table G.** Convergent Validity of the School Internal Engagement Scale-Refugee Parent (SIES-RP) Scale: Correlation matrix of scale items and estimates for items from CFA constructs (11 items, n=231).

| **School Internal Engagement Scale-Refugee Parent (SIES-RP) items retained** | 5.1.1 The interpreters available through the school are good  /effective | 5.1.3 It would be difficult for me to find a way to discuss an issue with school staff | 5.1.4 Despite the language restrictions I feel adequately informed about what happens at the school | 5.1.5 I feel that language restricts how much I can know about my child's progress | 5.1.6 I cannot be involved with the school as much as I would like because there are not enough speaking staff | 5.3.1 School staff direct me to services I need or that might be helpful to access | 5.3.3 The school helped or helps with the cost of uniforms or sports kit | 5.4.2 I regularly attend school events | 5.4.3 I have attended a multicultural day or event at the school, for e.g. Harmony Day | 5.4.4 I have attended parent-teacher interviews | 5.4.6 The school could do more to make me feel welcome and encouraged to participate | **Estimates from CFA** |
| --- | --- | --- | --- | --- | --- | --- | --- | --- | --- | --- | --- | --- |
| 5.1.1 The interpreters available through the school are good/effective | 1.00 |  |  |  |  |  |  |  |  |  |  | 0.37** |
| 5.1.3 It would be difficult for me to find a way to discuss an issue with school staff | 0.15* | 1.00 |  |  |  |  |  |  |  |  |  | 0.48** |
| 5.1.4 Despite the language restrictions I feel adequately informed about what happens at the school | 0.24** | 0.22** | 1.00 |  |  |  |  |  |  |  |  | 0.60** |
| 5.1.5 I feel that language restricts how much I can know about my child's progress | 0.05 | 0.25** | 0.26** | 1.00 |  |  |  |  |  |  |  | 0.45** |
| 5.1.6 I cannot be involved with the school as much as I would like because there are not enough XX-speaking staff | 0.26** | 0.42** | 0.29** | 0.30** | 1.00 |  |  |  |  |  |  | 0.60** |
| 5.3.1 School staff direct me to services I need or that might be helpful to access | 0.22** | 0.20** | 0.37** | 0.30** | 0.21** | 1.00 |  |  |  |  |  | 0.49** |
| 5.3.3 The school helped or helps with the cost of uniforms or sports kit | 0.22** | 0.23** | 0.06 | 0.09 | 0.19** | 0.15* | 1.00 |  |  |  |  | 0.27** |
| 5.4.2 I regularly attend school events | 0.04 | 0.07 | 0.19** | 0.12 | 0.09 | 0.10 | 0.07 | 1.00 |  |  |  | 0.27** |
| 5.4.3 I have attended a multicultural day or event at the school, for e.g. Harmony Day | 0.03 | 0.10* | 0.16* | 0.11 | 0.01 | 0.14* | 0.03 | 0.62** | 1.00 |  |  | 0.23** |
| 5.4.4 I have attended parent-teacher interviews | 0.06 | 0.01 | 0.22** | 0.17** | 0.02 | 0.10 | 0.03 | 0.21** | 0.18** | 1.00 |  | 0.19* |
| 5.4.6 The school could do more to make me feel welcome and encouraged to participate | 0.22 | 0.17* | 0.38** | 0.14* | 0.41** | 0.20** | 0.10 | 0.07 | 0.01 | 0.05 | 1.00 | 0.50** |
| Note: (*) and (**) indicate estimates statistically significant at p<0.05 and p<0.01. | | | | | | | | | | | | |

**Table H.** Convergent Validity of the School Community Engagement Scale-Refugee Parent School (SCS-RP) Scale: Correlation matrix of scale items and estimates for items from CFA constructs (7 items, n=231).

| **School Community Engagement Scale – Refugee Parent (SCES-RP) Scale items** | 5.5.1 The school has provided opportunities to meet and make friends with established Australians | 5.5.2 Through the school, I have met and made friends with people from similar backgrounds to me | 5.5.3 When I visit the school, I am likely to run into friends and acquaintances | 5.5.4 If I need advice about the school, I know other parents who I could call | 5.6.1 This school helps me feel like I belong in Australia | 5.6.2 Through the school, I have improved my English language skills | 5.6.3 Because of activities through the school (such as excursions) I better understand Australian customs | **Estimates from CFA** |
| --- | --- | --- | --- | --- | --- | --- | --- | --- |
| 5.5.1 The school has provided opportunities to meet and make friends with established Australians | 1.00 |  |  |  |  |  |  | 0.64** |
| 5.5.2 Through the school, I have met and made friends with people from similar backgrounds to me | 0.46** | 1.00 |  |  |  |  |  | 0.74** |
| 5.5.3 When I visit the school, I am likely to run into friends and acquaintances | 0.19** | 0.45** | 1.00 |  |  |  |  | 0.44** |
| 5.5.4 If I need advice about the school, I know other parents who I could call | 0.29** | 0.45** | 0.29** | 1.00 |  |  |  | 0.50** |
| 5.6.1 This school helps me feel like I belong in Australia | 0.18** | 0.18** | 0.17** | 0.08 | 1.00 |  |  | 0.25** |
| 5.6.2 Through the school, I have improved my English language skills | 0.43** | 0.39** | 0.15* | 0.26** | 0.08 | 1.00 |  | 0.59** |
| 5.6.3 Because of activities through the school (such as excursions) I better understand Australian customs | 0.41** | 0.29** | 0.14* | 0.11 | 0.18** | 0.48** | 1.00 | 0.50** |
| Note: (*) and (**) indicate estimates statistically significant at p<0.05 and p<0.01. | | | | | | | | |

**Table I.** Convergent Validity of School Belonging Scale-Refugee Parent (SBS-RP) Scale: Correlation matrix of scale items and estimates for items from CFA constructs (15 items, n=232).

|  | **School Belonging Scale – Refugee Parent (SBS-RP) items** | | | | | | | | | | | | | | |  |
| --- | --- | --- | --- | --- | --- | --- | --- | --- | --- | --- | --- | --- | --- | --- | --- | --- |
| **School Belonging Scale – Refugee Parent (SBS-RP) items** | 4.1 | 4.3 | 4.4 | 4.5 | 4.6 | 4.7 | 4.8 | 4.9 | 4.10 | 4.11 | 4.12 | 4.13 | 4.14 | 4.15 | 4.16 | **Estimates from CFA** |
| 4.1 I feel like a part of the school community | 1.00 |  |  |  |  |  |  |  |  |  |  |  |  |  |  | 0.64** |
| 4.3 It is hard for people like me to be accepted by school community | 0.20** | 1.00 |  |  |  |  |  |  |  |  |  |  |  |  |  | 0.33** |
| 4.4 Staff at the school take my opinions seriously | 0.27** | 0.10 | 1.00 |  |  |  |  |  |  |  |  |  |  |  |  | 0.44** |
| 4.5 Most teachers at school are interested in me | 0.51** | 0.25** | 0.44** | 1.00 |  |  |  |  |  |  |  |  |  |  |  | 0.61** |
| 4.6 Sometimes I feel as if I don't belong within the school community | 0.52** | 0.41** | 0.18* | 0.37** | 1.00 |  |  |  |  |  |  |  |  |  |  | 0.57** |
| 4.7 There is at least one teacher or staff member I can talk to at school if I have a problem | 0.28** | 0.08 | 0.18* | 0.29** | 0.21** | 1.00 |  |  |  |  |  |  |  |  |  | 0.48** |
| 4.8 People at school are friendly to me | 0.33** | 0.11 | 0.27** | 0.38** | 0.20** | 0.40** | 1.00 |  |  |  |  |  |  |  |  | 0.62** |
| 4.9Teachers here are not interested in people like me | 0.19* | 0.24** | 0.25** | 0.24** | 0.42** | 0.23** | 0.21** | 1.00 |  |  |  |  |  |  |  | 0.47** |
| 4.10 I am included in lots of activities at ___ school. | 0.12 | 0.13* | 0.05 | 0.02 | 0.18* | 0.01 | 0.05 | 0.12 | 1.00 |  |  |  |  |  |  | 0.15* |
| 4.11 I am treated with as much respect as other parents in the school | 0.27** | 0.16* | 0.16* | 0.27** | 0.21** | 0.40** | 0.44** | 0.24** | 0.05 | 1.00 |  |  |  |  |  | 0.54** |
| 4.12 I feel very different from most other people at school | 0.34** | 0.14* | 0.14* | 0.25** | 0.43** | 0.23** | 0.15* | 0.25** | 0.02 | 0.21** | 1.00 |  |  |  |  | 0.39** |
| 4.13 I can really be myself at school | 0.20** | 0.13 | 0.14* | 0.28** | 0.22** | 0.28** | 0.32** | 0.14* | 0.00 | 0.33** | 0.29** | 1.00 |  |  |  | 0.42** |
| 4.14 Teachers at school respect me | 0.23** | 0.21** | 0.26** | 0.37** | 0.20** | 0.36** | 0.50** | 0.25** | 0.12 | 0.46** | 0.13 | 0.36** | 1.00 |  |  | 0.59** |
| 4.15 I wish that my family and I were involved in/ attended a different school in Australia | 0.35** | 0.19* | 0.29** | 0.22** | 0.33** | 0.21** | 0.35** | 0.36** | 0.10 | 0.28** | 0.12 | 0.12 | 0.33** | 1.00 |  | 0.57** |
| 4.16 I feel proud to be a part of the school community | 0.54** | 0.17* | 0.28** | 0.38** | 0.37** | 0.24** | 0.45** | 0.32** | 0.17* | 0.34** | 0.18* | 0.21** | 0.40** | 0.58** | 1.00 | 0.70** |
| Note: (*) and (**) indicate estimates statistically significant at p<0.05 and p<0.01. | | | | | | | | | | | | | | | | |

**Table J.** Convergent Validity of Cultural Broker Relationship Scale (CBRS): Correlation matrix of scale items and estimates for items from CFA constructs (5 items, n=216).

| **Cultural Broker Relationship Scale (CBRS) items** | 5.2.1 XX has been really important in making me feel secure in the school because xx understands my family's religious and cultural backgrounds | 5.2.2 When we first enrolled at this school, I felt comfortable because xx was there to assist us | 5.2.3 I can contact xx if I need to | 5.2.5 I only feel comfortable meeting with other staff, like the principal or classroom teacher, if xx is there | 5.2.6 If it weren't for xx I would not know how to engage with the school | **Estimates from CFA** |
| --- | --- | --- | --- | --- | --- | --- |
| 5.2.1 XX has been important in making me feel secure in the school because xx understands my family's religious and cultural backgrounds | 1.00 |  |  |  |  | 0.73** |
| 5.2.2 When we first enrolled at this school, I felt comfortable because xx was there to assist us | 0.60** | 1.00 |  |  |  | 0.80** |
| 5.2.3 I can contact xx if I need to | 0.26** | 0.37** | 1.00 |  |  | 0.42** |
| 5.2.5 I only feel comfortable meeting with other staff, like the principal or classroom teacher, if xx is there | 0.33** | 0.31** | 0.18* | 1.00 |  | 0.46** |
| 5.2.6 If it weren't for xx I would not know how to engage with the school | 0.20** | 0.24** | 0.05 | 0.49** | 1.00 | 0.34** |
| Note: (*) and (**) indicate estimates statistically significant at p<0.05 and p<0.01. | | | | | | |
