# Supplementary material for: Psychological distress, resettlement stress, and lower school engagement among Arabic-speaking refugee parents in Sydney, Australia: A cross-sectional cohort study
Source: PLoS Med. 2021 Jul 12;18(7):e1003512. doi: 10.1371/journal.pmed.1003512 (PMC8312975; doi:10.1371/journal.pmed.1003512)
Supplement: Attachment — Submitted filename: ResponsetoEditors.PLOSMedicine.29.7.20.FINAL.docx [file pmed.1003512.s004.docx]

**Response to Editors and Reviewers**

**Requests from the editors:**

**Comment 1:** I understand the paper was submitted for the journal's special Collection on Refugee and Migrant Health; that collection has now launched

(<https://collections.plos.org/refugee-health-special-issue>) so the paper you've submitted is too

late for the launch. However, it can still be considered for the journal and if it passes the journal's usual criteria, published; we'd also hope it can subsequently included in the collection's homepage as well.

**Response:** Thank you for your continued consideration in PLOS Medicine.

**Comment 2:** Please revise the title according to PLOS Medicine's style. Your title must be nondeclarative and not a question. It should begin with main concept if possible. Please place the study design in the subtitle (ie, after a colon) - eg, here, "xxyy: cross-sectional study".

**Response:** We have amended the title to include the study design as per below:

*“Psychological distress and resettlement stress are associated with lower school engagement among Arabic-speaking refugee parents:* a cross-sectional cohort study*”*

**Comment 3:** At this stage, we ask that you include a short, non-technical Author Summary of your research to make findings accessible to a wide audience that includes both scientists and non-scientists. The Author Summary should immediately follow the Abstract in your revised manuscript. This text is subject to editorial change and should be distinct from the scientific abstract. Please see our author guidelines for more information: <https://journals.plos.org/plosmedicine/s/revising-your-manuscript#loc-author-summary>

**Response:** This has been written and included in text, as per below:

***Why Was This Study Done? Schools are more than just places to learn for many resettled refugee youth. Evidence suggests that they are also places to support refugee families’ sense of belonging and integration into the community. This study was undertaken to quantify key aspects of refugee parents’ school involvement, beyond which existing measures of parent school engagement are able to capture.***

***What Did the Researchers Do and Find?*** *Using data from a cohort study of 233 Arabic-speaking refugee families, the study developed and validated four self-reports tools to assess parent school engagement and affective sense of belonging. This included measurement of the quality of the relationship between parents and the bilingual officer often employed by schools to act as a cultural broker. Secondary analyses found that parents’ experiencing psychological distress or post-migration living difficulties were less engaged with their child’s school.*

***What Do These Findings Mean?*** *These new refugee-specific measures of parent engagement provide a resource to evaluate government efforts to support the integration of refugee families into schools. The cross-sectional design precludes casual interpretations. Nonetheless, the findings speak to the implementation of initiatives that consider the identification and capacity-building of parents who are experiencing psychological distress or resettlement stressors.*

**Comment 4:** Currently, the abstract states that one aim of the work is to "...measure the effectiveness of school-based parent engagement initiatives" - I wasn't clear that this was something that the current study design was capable of doing (given that the study doesn't seem designed to assess the effects of an intervention(s) per se - eg, such as using RCT design) - perhaps this can be considered and the explanation of aims framed a bit more clearly?

**Response:** This statement was ambiguous. We have now framed it to read more clearly as:

*“The study first aimed to validate self-report measures of parent school engagement developed specifically for the refugee context; and second, to identify socio-demographic*

*parent characteristics that may inhibit school engagement, so as to help tailor support to families most in need.”*

**Comment 5**: Ideally, please rework the in-text referencing style (this should be simple if referencing software is used) to use sequential numbering in square (not round) brackets. Many thanks.

**Response:** Amended

**Comment 6: a)** Can you clarify in the Methods section if the study had a prospective protocol or analysis plan? Please state this (either way) early in the Methods section.

**b)** If a prospective analysis plan (from your funding proposal, IRB or other ethics committee submission, study protocol, or other planning document written before analyzing the data) was used in designing the study, please include the relevant prospectively written document with your revised manuscript as a Supporting Information file to be published alongside your study, and cite it in the Methods section. A legend for this file should be included at the end of your manuscript.

**c)** If no such document exists, please make sure that the Methods section transparently describes when analyses were planned, and when/why any data-driven changes to analyses took place.

**d)** In either case, changes in the analysis-- including those made in response to peer review comments-- should be identified as such in the Methods section of the paper, with rationale.

**Response:** Amended. We have added this information, and referred to the supporting document, in the Design subsection of the Methods as follows:

*The study had a prospective protocol, which was approved by the University’s Human Research Ethics Committee (HC15833) and State Department of Education (SERAP 2016056) in advance of the study commencing (see supporting information file).*

We also note in a footnote on p.15 that “The original intention was to conduct linear regression analyses. This plan was modified at the peer-review stage to involve stepwise (forward) regression analyses.

**Comment:** *At the moment, figure 1 is named (in the title and also the in-text callouts) as a CONSORT flow diagram. CONSORT is the reporting tool normally used for randomized trials; the actual figure is fine as it is, but I'd suggest renaming the flow diagram of recruitment, this could just simply say (in title and text) "Flow diagram of Parent/Participant recriutment".

**Response:** All references to the CONSORT diagram (in title and in-text callouts) have been amended to “Flow diagram of Parent Participant Recruitment”.

**Comment:** *We would suggest ensuring that the study is reported according to the STROBE guideline (recommended for observational studies - case/control, cohort, and cross-sectional studies), and include the completed STROBE checklist as Supporting Information. Please add the following statement, or similar, to the Methods: "This study is reported as per the Strengthening the Reporting of Observational Studies in Epidemiology (STROBE) guideline (SChecklist)." The STROBE guideline can be found here: <http://www.equator-network.org/reporting-guidelines/strobe/>. When completing the checklist, please use section and paragraph numbers, rather than page numbers.

**Response:** We have completed the STROBE checklist. It is referred to in the Design subsection (and legend) as a supporting file. In addition, extra text has been added to the paper as per the STROBE guidelines. These include more information on missing data in table footnotes, plus the following text:

- *A standardised approach with a defined recruitment protocol and eligibility criteria was implemented to minimise bias (Design subsection)*
- *The study was conducted at five primary schools in a low socio-economic multicultural community in Sydney, Australia in 2016 (Setting subsection)*
- *Monte Carlo simulations estimated that, for coefficients of 0.5, 80 parent-child dyads per school would be sufficient to reject the null hypothesis at the 0.05 probability level, applying a two-tailed test and a critical value of 1.96. (Participants subsection)*
- *The anticipated resettlement of 12,000 refugee families into the local area was delayed, thus the sample size was lower than prospective estimates detailed earlier. (Sample Characteristics subsection)*

-----------------------------------------------------------

Comments from the reviewers:

Reviewer #1: I confine my remarks to statistical aspects of this paper. While the general approach is appropriate, I have a number of issues to resolve before I can recommend publication

**Comment:** Line 337 Why was 85% chosen? This may or may not be a good choice, depending on what it retains and eliminates. Sometimes it is good to have one or two items with a ceiling, as some respondents may respond "correctly" only to that item. ("Correctly" is not quite right, but really means "most positive way")

Line 338-340 Why remove these items before the CFA? The CFA ought to indicate that they are not useful.

**Response:** Thank you for raising this issue. In the revised analysis we have included all the specific sub-scale items in the CFA. Statistically significant items (p<0.05) in CFA were retained for their respective scale. In the revised analyses, the CFA findings guided which items were retained for the final scales, and the same rules applied across sub-scales.

**Comment:** Lines 356-358. This approach, known as bivariate screening, cannot be recommended. The resulting multiple regression will be wrong in several ways: Standard errors will be too small and, as a result, p values will be too low and CIs too narrow. In addition, the parameter estimates will be biased away from 0. It is best to use substantive knowledge to build a model, but if the authors insist on using an automatic method, then LASSO is usable.

**Response:** As per reviewer comments, we have included all theoretically relevant predictors in the stepwise multiple regression analysis. Standardised regression co-efficients for significant predictors from the stepwise forward linear multiple regression analyses are presented in Table 6. Bivariate associations of all theoretically relevant predictors with mean scale scores and p-values from statistical tests are presented in Table 5. In a situation where measurement units for predictor variables differ from one another, standardised regression co-efficients are an appropriate and commonly used option. If a predictor is not found to be significant in bivariate analyses, then it is typically not found to be statistically significant in multiple regression models, in the presence of all other predictors in the model. As expected, those predictors not found to be statistically significant in bivariate analyses, were not significant in the multiple regression analyses. The variables found to be significant in bivariate analyses are also significant (p<0.05) in the stepwise multiple regression analysis. This consistency of findings indicates that multiple regression analysis is appropriate for our data.

Regarding standard error and p-values, we are unfamiliar with guidelines that multiple regression analysis cannot be used when standard error or p-values are smaller than one. In any type of multivariate analysis, standard error for the predictor depends on sample size (i.e. for a large sample standard error will be smaller); and p-values are dependent on both sample size and sub-group differences. In SPSS, stepwise forward linear multiple regression analysis technique is similar to LASSO (Least Absolute Shrinkage and Selection Operator) forward technique. Since the term Multiple Regression is more familiar to readers, relative to LASSO, we have stayed with stepwise forward multiple regression as the analysis of choice. (However, if the reviewers feel strongly about this, we are open to change the name to LASSO linear regression (forward).

**Comment:** Line 358-359 What exactly does this mean? Categorizing continuous variables is a mistake; it leads to an increase in both type I and type II error. If there are outliers in an IV, they can be dealt with by choice of method (e.g. robust regression).

**Response:** All our outcome variables are continuous. For multiple regression analyses we treated all the continuous variables as continuous. We have revised this to make this clearer throughout the measures section and as footnote in Table 6. In the revised bivariate analyses, along with categorisation, we also present the correlation co-efficients between continuous predictors and scale scores. The findings are consistent with what we found in the categorical presentation. To be consistent with other dichotomous and categorical predictor variables, we categorised continuous predictor variables. In bivariate analyses, categorisation of continuous predictor variables is common, e.g. age group, income group, psychological distress, BMI index etc. There were no outliers in any of our IVs. For our continuous IVs, the range of both skewness and kurtosis are within the acceptable range of between +2 and −2 (Skewness: ranges 0.013 to 0.87; Kurtosis ranges -0.29 and 0.68).

**Comment:** Line 359=360 Colinearity can occur between more than two variables. What was done then?

**Response:** In the revised manuscript, we have used stepwise forward multiple regression technique. During the estimation process this technique automatically removes the variable by considering the value correlation and collinearity statistics tolerance. We have revised the text throughout to accommodate this change.

**Comment:** Line 361-363 You cannot accept the null, only fail to reject. If the authors want to do equivalence testing, they can, but that would be a separate procedure.

**Response:** We have removed this comment in line with the revised analysis.

**Comment:** Line 365-367	I don't understand this. What was done?

**Response:** We have deleted this sentence.

-----------------------------------------------------------

Reviewer #2: Review of PMEDICINE-D-19-03661 Psychological distress and resettlement stress are associated with lower school engagement among refugee parents

The purpose of the paper is to develop culturally-valid measures of school engagement among refugee parents in Australia and to describe the association of school engagement with other sociodemographic characteristics and psychological states of refugee parents. I very much appreciate the careful attention these authors give to establishing measures appropriate to refugee populations. It is important to not impose etic constructs.

Theoretical and Framing Issues

**Comment 1**: The paper needs more theoretical development. I understand that the qualitative research findings are reserved for another paper. However, there needs to be a discussion of the literature on parental engagement, which is broader and more sophisticated than the authors' suggest with their statement that existing engagement measures focus primarily on behavioral engagement. Several dimensions of parental/family engagement have been identified in addition to behavioral supports of student learning as evidenced by participation (which I agree is the most common). These include perceptions that the school is a welcoming environment, effective communication with school staff, and sharing power an advocacy, among others. The paper needs a review of these existing constructs and describe how the qualitative data showed that additional dimensions of school engagement are relevant for Arab-speaking refugees or that existing measures do not adequately capture the construct for Arab-speaking refugees. The new measures should fill this void, and predictive and discriminant validity established by testing the association with existing engagement measures. This was done to a limited extent with the Delaware scale, but it all seemed a bit random because of the lack of a theoretical framework.

**Response:** Bar the first paragraph, the introduction has been restructured and rewritten to provide a more theoretical framework, as well as a present a clearer case for the inadequacy of existing parent engagement measures in relation to refugee families and why additional measures are required. References are now provided to support the choice of the Delaware scale:

*“Several theories of parent school engagement exist [15-17]. The most common and comprehensive framework is Epstein’s six different types of parent involvement [16]. The first type of involvement is parenting and discusses the school’s role in educating families on how to create a positive home environment. The second type of involvement is centered on effective communication between home and school. The other types include recruiting parents to volunteer at the school, involving parents in school decision-making, home learning, and community collaborations.*

*These traditional definitions of parental school involvement may not adequately capture engagement as it applies to refugee families [18]. Volunteering or supporting children with learning at home are compromised by many refugees’ limited English proficiency [19-23], as well as time constraints related to work and economic survival and a need to focus on the family’s core needs [19, 20, 23, 24]. Past disruptions in schooling during displacement, and limited experience with school systems in countries of resettlement may also make typical parent involvement difficult [24, 25]. Qualitative research suggests cultural ambiguity between expectations of parents and the school regarding what constitutes “good” school engagement. For instance, findings that refugee parents express concerns that it may be culturally inappropriate to “interfere” with the work of teachers, and keeping ones distance shows the reverence and high esteem they hold for schools as an institution, has implications for the decision-making facets of parent involvement [26].*

*Indeed, the high value refugee parents place on their child’s education may be expressed in different ways [23]. For example, whilst not focused on refugee children, a study of a large national student sample in the U.S. found that academic achievement amongst White American families was positively associated with parent participation in school-based activities; however, for Asian American families the relationship was inverse [27]. In another study, ethnicity was found to influence the relationship between 12 types of parent involvement and student achievement [28].*

*Moreover, the seeming bias towards more behavioural indicators of parent involvement [15-17], neglects the relational or more affective component implicit in parent involvement. In the refugee field, parents’ wellbeing includes the psychological effects of past trauma and displacement [29] and distrust of authorities based on past experiences of persecution and discrimination [30]. The extent to which refugees can establish relationships of trust and safety may therefore be foundational to any worthwhile engagement with schools. For example, in a study of Hmong refugees in the U.S., a school ethos of inclusion was found to be the first step in removing parents’ expectations of deference towards any “authority” [23].Furthermore,* *numerous case studies have showcased how adept certain schools and their staff have become at engaging with refugee families [31, 32]. Current measures of parent school involvement may not capture this [33]. For instance,*

*schools have been found to play an instrumental role in guiding parents to local support agencies and helping them navigate unfamiliar and often overwhelming government systems [31, 35]. Similarly, reports of social activities at the school, such as breakfast clubs and diversity days have been posited to connect parents with each other, whilst school-initiated parent classes in the region’s primary language have been documented to improve cultural competency [36]. A key piece in this literature is the consistent role that bilingual school liaison officers play in facilitating all these processes [6, 23, 26, 37]. Such persons are employed by the school, specifically tasked with brokering the relationship between refugee families and the school, as well as the wider community. To date, no measure exists to capture this influential relationship between the cultural broker and parents.*

*The development of such measures is important. Several countries such as the UK, USA, Canada, Australia, Sweden, and Germany have government policies and programs in place to support the integration of refugee families into schools. Targeted measures of impact that capture the distinct ways in which refugee parents might engage with schools are needed to track the success of these initiatives. Context-specific measures also carry important implications for advancing efforts to improve refugee parents’ engagement in their children’s schooling in a tailored way that is most valuable to the family. Teasing out the nuances of refugee parent school involvement relative to non-refugee families, also has the benefit of avoiding any misguided findings that refugee families might be less invested in their children’s education.*

*To this end, the primary aim of the present study was to develop and validate self-**report tools to measure refugee parents’ involvement in school - in a traditional sense, as well as the “extra” targeted elements of engagement that foster families’ social and cultural capital. The study also aimed to develop and validate a self-report tool to capture an affective index of engagement - namely parents’ sense of belonging or feelings of connectedness or closeness to the school - and ultimately, the vital relationship between refugee parents and the schools’ cultural broker. An established measure of teacher-home communication, a robust facet of parent involvement, was selected as a distal measure of convergent validity [39-41].*

*The secondary aim of the present study was to then identify refugee parent characteristics associated with these new measures of parent involvement. Whilst some refugee families may engage readily and easily with school and not require special assistance, other families may have the opposite tendency and require more active assistance. Profiling refugee parents at “risk” of low levels of engagement can help schools to individualise or tailor their support to families most in need. It was reasoned that these factors would likely mirror some of the barriers to traditional engagement described earlier [19-24]. For instance, in the migrant literature, low socioeconomic status, less education and time preoccupation with daily environmental stressors have been associated with lower parent engagement [42-48].*

*Specifically, it was hypothesised that countervailing parental factors such as length of time lived in the host country, employment and visa status, psychological health, English language proficiency, trauma experience and post-migration stressors would be associated with school engagement and belonging.”*

The absence of measures to test predictive and discriminant validity is noted as a limitation in the first paragraph of the discussion, as per below

*“Future research is required to separate out the nuances of each measure with distinct measures of validity, including predictive and divergent validity*.”

**Comment:** The items developed for the scales do not follow best practices. They include reverse-coded items. The questions are complex, which makes it difficult to interpret the answers. Some questions combine parental engagement and barriers to parental engagement in a single question. For example, "Despite the language restrictions I feel adequately informed about what happens at the school." This question assumes that parents experience language restrictions, whereas your data show that many do not. If I don't experience language restrictions, how do I answer this question? Other questions are double-barreled. For example, "XX has been important in making me feel secure in the school because XX understands my family's religious and cultural backgrounds." What if XX understands family background but does not make them feel secure in school? These should have been separate questions.

**Response:** We heed the reviewer’s concerns about the structure of the questionnaire items. Unfortunately, it is not possible to change the items retrospectively without jeopardising the study. We acknowledge this significant limitation in the first paragraph of the discussion. We also acknowledge that the measures still performed okay as a whole. Items that did not perform well were ‘washed out’ by the ones that did, and the final items still cohered in a factorial analysis. The questionnaire items were read out in an interview format, so this may have assisted with their comprehension.“*Whilst a couple of the questionnaire items were double-barrelled and could have been interpreted ambiguously, all measures indicated acceptable reliability [67] and sound construct validity”*

**Comment:** The scales contain items that on their face seem to measure distinct dimensions of parental engagement. For example, the individual engagement scale includes questions about financial support, parental behavioral involvement, communication, and language barriers. In the larger literature, as noted above, these are treated as separate dimensions of engagement. One of the limitations of the current research on parental engagement, which also suffers from this type of fuzzy measurement, is that it does not lend itself to guiding interventions. Which of these aspects should schools focus on? Which is foundational for the others to be present? Ken Bollen (1990) has an excellent discussion of how highly correlated items may still represent distinct dimensions of community engagement.

**Response:** This is an extremely valid point and one that could possibly be subsumed under the wider debate of nomothetic grouping for parsimony and generalisation, versus a more idiographic approach. The paper feels quite dense already with the development (and ensuing analyses) of four distinct new measures. We were concerned that to break the scales down further into subscales may be somewhat overwhelming and beyond the scope of the paper. Theoretically and statistically, it felt more meaningful to separate the scales by internal (communication, assistance, and parent engagement) and community engagement (social capital and acculturation). However, future use of the individual items to guide specific and foundational interventions is noted in the penultimate paragraph of the discussion, as per below.

*“Individual item-predictor analysis would also be helpful in guiding interventions and pinpointing exactly which tangible dimensions of engagement schools should be focusing on^[1]^. For instance, during the scale development, it was pertinent that items related to effective communication correlated strongly with parent engagement items, and items related to social capital correlated strongly with acculturation items.”*

**Comment:** Concurrent validity, as measured by correlations with the THC, needed some discussion.

**Response:** Concurrent validity has been given more attention in the opening paragraph of the discussion, as per below:

*“Convergent validity was based on positive correlations with a measure of teacher-home communication. It makes sense that the strongest correlations were with the measures assessing sense of belonging and internal school engagement (SBS-RP and SIES-RP), relative to the SCES-RP and CBRS which encompass outside community factors or a relationship independent of the teacher and an ability to speak English.”*

**Comment:** Finally, the latter part of the paper and the discussion is too causal in nature, even though it is acknowledged that the data are cross-sectional. It presumes a unidirectional association that poor parental mental health reduces family engagement. Research suggests a bidirectional relationship such that that family engagement is necessary for schools to reach parents to improve mental health of both children and parents. See, for example, McNeely et al. (2020; Journal of School Health) and Ellis and colleagues (e.g., Kia-Keating & Ellis, 2007; Clinical Child Psychiatry and Psychology).

**Response:** We have amended the discussion to soften the causality, e.g. reworded “predicted” as “associated with”. We have also emphasised the bidirectional nature of the findings, as per below:

*“The finding also aligns with bidirectional research - that family engagement is necessary for schools to be able to reach parents to help improve their and their child’s mental health [68, 69]. Certainly, noting the correlational study design, parents with mental health issues may be harder to engage or feel more disconnected.”*

Methodological Suggestions

**Comment a)** The authors drop variables due to their data distribution or missing data. They did not say which variables were excluded or how many, and I was left wondering if these variables were conceptually important. Fortunately, Mplus can handle variables with missing data. The variables with ceiling effects can be treated as categorical variables in the CFA model. Given that the authors chose CFA over EFA, I suspect that all of the variables used to measure the latent constructs have theoretical importance and should be included if possible.

**b)** The authors also drop variables because they have low factor loadings. What are these variables? Do they form a different, also important dimension of social belonging or did they have low factor loadings due to measurement issues (e.g., confusing wording, reverse scoring)? This gets back to the issue of having a theoretical basis for selecting the items and an empirical basis for dropping them.

**Response:** Thank you for your comments. This issue has been raised by another reviewer also. As above, in the revised analyses none of the items were arbitrarily dropped – the CFA findings guided which items were retained. We have included all the scale items in the CFA. We did not drop any of the variables due to their data distribution or missing data. Items found to be statistically significant (p<0.05) in the CFA are retained for their respective scale. The labels of all specific items for each scale measure, including those retained or dropped through CFA, are presented in supporting information files S3 and S4.

**Comment:** It would be valuable to test for factor invariance across groups, particularly between early arrivers and long-term residents/citizens in Australia. Not sure if there is enough statistical power, but could the authors explore this? This would be particularly useful given the unexpected finding that duration of time in Australia is associated with lower school engagement. One potential explanation is that the schools are so geared towards serving newcomers that long-term residents feel less engaged.

**Response:** Thank you for your suggestions. In Table 5, we presented the mean scale scores for three broad lengths of duration subgroups, and F-tests (ANOVA) were used to see the differences of scale scores, and p-values from F-test also included. We agree that duration is an important factor. Unfortunately, due to small sample size across various lengths of duration which ranges from 3 months to a stay of 21 years in Australia, a test of invariance by various duration lengths may not produce reliable estimates. We speak to this study limitation and other potential explanation in the discussion as per below:

*“The small sample size across a wide duration of stay from 3 months to 21 years, unfortunately prohibited comparisons between early arrivers and long-term residents in Australia.”*

*“One potential explanation, similar to the cultural broker reasoning detailed above, is that schools are so geared towards serving newcomers that long-term residents feel less engaged^[2]^.”*

Presentation Suggestions

**Title:** Might you consider replacing the term "refugee parents" with "Arabic-speaking refugee parents" in the title. Refugees are an extremely heterogeneous group.

**Response:** We have amended the title to read as: “*Psychological distress and resettlement stress are associated with lower school engagement among Arabic-speaking refugee parents: A cross-sectional cohort study”*

**Comment:** Essential information about some of the scales is missing in the measures section. How many items are on the Delaware school climate survey teacher-home communication subscale and the PMLD scale. Also, I'm not sure it makes sense to report the reliability of the number of trauma exposures because this is a count variable. Measures of reliability assume that all the variables in your scale measure the same underlying construct, which is not the goal in counting the frequency of different traumatic experiences to determine the level of adversity exposure.

**Response:** Number of items (4) on the Teacher-Home communication subscale, and on the PMLD (28) have been added in-text. Trauma count is not a single item variable. The trauma count was generated based on trauma related items with a Yes or No response. For dichotomous data Kuder-Richardson (KR-20) coefficient is commonly used and we took the opportunity to report KR-20 for our data.

**Comment:** Items of the new scales are only reported in the supplemental index, which will make it difficult for readers to access. These should be reported in the main paper.

**Response:** We understand that this would be ideal. However, there are several tables already included within the text. When we trialled adding tables of the scale items within the text too, the paper felt quite unwieldy. For this reason, we have included the tables listing all final items of each scale in the supplemental index. If the reviewers feel strongly about this, we are open to re-include them.

**Comment:**

The supplemental index is confusing. The first table, presenting a correlation matrix, looks really interesting. But you list scales that you never present in the paper. I'm wondering if these scales wouldn't be more appropriate since they seem to distinguish dimensions of engagement more clearly. They also show very interesting findings, which are consistent with qualitative research and would be useful for policy, e.g., that for refugee parents, perceived quality of communication is highly correlated with assistance from the schools. That kind of specificity is needed for schools to know what to do.

**Response:** Thank you for your suggestion. We have reordered the tables consistent with the order of analyses. We agree that the scales hold interesting and useful information – communication seems key. Acculturation and social capital are highly correlated which also makes meaningful sense. However, for parsimony in an already dense paper, we are waiting to explore this data in-depth in a separate paper. We note this future research potential in the penultimate paragraph in the discussion, similar to an earlier suggestion:

*“Individual item-predictor analysis would also be helpful in guiding interventions and pinpointing exactly which tangible dimensions of engagement schools should be focusing on^[3]^. For instance, during the scale development, it was pertinent that items related to effective communication correlated strongly with parent engagement items, and items related to social capital correlated strongly with acculturation items.”*

**Comment:**

The supplemental tables showing item-to-item correlations uses the variable names instead of the variable labels, making it difficult to read. I had to assume the variables were listed in the same order as they were in the prior table. By "estimates from CFA," did you mean the factor loadings? In these tables it would also be interesting to have an additional column reporting the Chronbach's alpha for the scale if the item had been dropped.

**Response:** In the revised Supplementary files we have included descriptions of all items. In Table 2, we have added values Cronbach's Alpha (α) for each model. We have also added values for Akaike Information Criterion (AIC) and sample size adjusted Bayesian Information Criterion (BIC) for each model. The values of Cronbach's Alpha (α), AIC, BIC and other summary statistics shows clear improvement of the final models after dropping the insignificant items, as compared to before exclusion of those items.

-----------------------------------------------------------

Reviewer #3: This is a well-done study guided by theory with community implications.

**Comment:** The lack of more details about the translation process is a weakness and should be expanded or listed as a weakness.

**Response:** We have described the limitations of this approach in the discussion:

*“An asset of the study was that refugee families were able to be interviewed in their native language. This diverges from many culturally diverse studies that often require English language proficiency, lending itself to a biased, potentially more acculturated, sample. However, not using accredited translators limits the reliability of the measures.*

**Comment:** Also, the lack of analysis incorporating school as a cluster was disappointing. A statistician should review and make suggestions. Other than those criticisms, it is a well-done, well-written study.

**Response:** We have included scale scores for enrolled school in the revised analyses. The findings show that schools differed by internal engagement. Sample size for each school (ranges n=33 to n=60) was not large enough to look at clustering. We have highlighted issues related to this in the discussion:

*“School site was not controlled for in the analyses, and as such school-level factors are something to consider with cluster-analysis in the future [76, 77]. This is especially so, given that internal school engagement was shown to vary across schools.*

1. Thank you to an anonymous reviewer for this suggestion
2. Thank you to an anonymous reviewer for this interpretation.
3. Thank you to an anonymous reviewer for this suggestion
