# Supplementary material for: Psychological distress, resettlement stress, and lower school engagement among Arabic-speaking refugee parents in Sydney, Australia: A cross-sectional cohort study
Source: PLoS Med. 2021 Jul 12;18(7):e1003512. doi: 10.1371/journal.pmed.1003512 (PMC8312975; doi:10.1371/journal.pmed.1003512)
Supplement: Attachment — Submitted filename: 2ndResponsetoReviewers.FINAL.27.9.20.docx [file pmed.1003512.s005.docx]

**Response Reviewers**

**Reviewer #1:**

**Comment 1:**

The authors wrote <<<If a predictor is not found to be significant in bivariate analyses, then it is typically not found to be statistically significant in multiple regression models, in the presence of all other predictors in the model.  As expected, those predictors not found to be statistically significant in bivariate analyses, were not significant in the multiple regression analyses. The variables found to be significant in bivariate analyses are also significant (p<0.05) in the stepwise multiple regression analysis. This consistency of findings indicates that multiple regression analysis is appropriate for our
data.>>>

This is not always the case and it overemphasizes statistical significance. The results of stepwise methods are wrong. As I said, p values are too low, standard errors too small, parameter estimates biased away from 0.
Results from stepwise and bivariate analyses should not be presented.

The fact that none switched from significant to non-sig., or vice versa, is not really important.
The authors wrote <<< In SPSS, stepwise forward linear multiple regression analysis technique is similar to LASSO (Least Absolute Shrinkage and Selection Operator) forward technique. Since the term Multiple Regression is more familiar to readers, relative to LASSO, we have stayed with stepwise forward multiple regression as the analysis of choice. (However, if the reviewers feel strongly about this, we are open to change the name to LASSO linear regression (forward).>>>

It's not a question of the name, but the whole analysis. The results from LASSO should be presented. The fact that it is less familiar isn't really important - maybe some readers will learn something.

**Response:** Thank you for your comments. We have removed the bivariate analyses in the revised manuscript. Instead of stepwise regression, we have used LASSO (Least Absolute Shrinkage and Selection Operator) Regression analysis. LASSO regression was performed by SAS 9.4 software [PROC GLMSELECT with selection=forward (select=SL stop=PRESS (predicted residual sum of squares)]. LASSO regression estimates are presented in revised manuscript Table 6 and Table 6a.

**Comment 2:**

The authors wrote: <<<To be consistent with other dichotomous and categorical predictor variables, we categorised continuous predictor variables. In bivariate analyses, categorisation of continuous predictor variables is common, e.g. age group, income group, psychological distress, BMI index etc.>>>

It's certainly common to do this but it is a mistake to do so.

**Response:** Bivariate analyses are now excluded. Continuous predictor variables are included in the LASSO regression in their continuous format (Table 6 and 6a).

**Comment 3:** The authors wrote <<< In the revised manuscript, we have used stepwise forward multiple regression technique. During the estimation process this technique automatically removes the variable by considering the value correlation and collinearity statistics tolerance. We have revised the text throughout to accommodate this change>>>

This is not correct. See e,g. this thread  <https://stats.stackexchange.com/questions/186081/is-multicollinearity-an-issue-when-doing-stepwise-logistic-regression-using-aic>
What happens is that SPSS will make an essentially arbitrary choice among colinear variables. This won't affect prediction, but it can lead to very poor explanation and to wrong conclusions about which variables are important.

**Response:** Thank you for your comments and attached URL about multicollinearity. In the revised analysis, we used variance inflation factor (VIF) and tolerance statistics to examine the multicollinearity among all predictors accounting for each outcome factor. The value of VIF for predictor variables ranged from 1.14 to 2.25; the maximum VIF value 2.25 is not substantially greater than 1.0 and is less than 10. Multicollinearity tolerance statistics ranges from 0.44 to 0.87; the minimum tolerance statistics value 0.44 is greater than 0.10. Although some of our predictor variables are significantly correlated one another, the maximum value of correlation 0.48 is less than 0.70; the maximum VIF value 2.25 is less than 10 and minimum value of tolerance statistics 0.44 is greater than 0.10; indicating that there is no multicollinearity exists among predictors entered in LASSO regression. We have highlighted this multicollinearity comment, in the predictive analyses sections of the methods and results, as per below:

*“Prior to conducting LASSO regression, we used variance inflation factor (VIF) and tolerance statistics to examine the multicollinearity among the predictors accounting for each outcome factor.”*

*“The VIF for the predictor variables ranged from 1.14 to 2.25. Multicollinearity tolerance statistics ranged from 0.44 to 0.87. Whilst some predictor variables were significantly correlated with one another, the maximum value of correlation coefficient r=0.48 was less than <0.70; the maximum VIF value of 2.25 was less than 10 and the minimum tolerance statistic of 0.44 was greater than 0.10. This indicated that there was no remarkable multicollinearity among predictors entered in the LASSO regression.”*

**Reviewer #2:**

Thank you for attending to my concerns about the lack of justification for the study and the lack of a theoretical framework. The revisions to the introduction helped to address these concerns. The rewriting of the discussion also helped.

**Comment 1:** Unfortunately, the methodological approach used still does no align with the new theoretical development presented and is still incomplete. Content Design and Development of New Measures: I found the formatting of this section confusing. I think it is an easy fix. Isn't the determination of these measures the result of the paper? Yet you describe the measures as if they are already validated. I had expected to see a description of your process for organizing the multiple items on the master list into theoretically distinct constructs as laid out in the intro. This is a major part of the methods that you entirely skip over. Was EFA used for this? Another process? Please describe.  Also, please provide information on whether the items were originally written in English or Arabic and, regardless, if they were translated and back-translated.

**Response:** Thank you for this. We hope that the readability has significantly improved, by, as per suggested, placing the measures and their development into the results section. We have also tried to break the development down into parts, and the scales only referred to as scales after the CFA, to try to delineate the steps in the process more; see the “Item Development and Pilot” section on pp. 16-18 focusing on the creation of the measure items, followed by the section “Confirmatory Analyses” on pp.18-19 indicating how the final items were selected and the production of the four final scales). We have also expanded further on the pilot process as per below.

*“Based on the desk review and qualitative study, authors JB and DH generated a pool of possible items written in English measuring school engagement. These were reviewed and edited for content and cultural appropriateness by a local primary school teacher and author AA (an active member of the local Arabic-speaking community with excellent English language skills). Author AA translated the items into Arabic, which were assessed for comprehension and cultural applicability by Arabic members of the research team.*

*The items were piloted with several parents enrolled in the study, according to the interview protocol. A few items were dropped due to ambiguity, where the interviewee had to explain the item further, or where the parents interpreted the items in different ways during administration. Based on this feedback and a roundtable discussion, consensus was reached on a final master list of items that was translated into Arabic to form a provisional set of school engagement items , a provisional set of items pertaining to the cultural broker-parent relationship, and a provisional set of items pertaining to parent sense of school belonging, as detailed below.”*

The items were not formally back translated. This is acknowledged as a limitation in the discussion: *“However, not using accredited translators and the omission of backward translation limits the reliability of the measures.”*

**Comment 2:** Line 309: What does it mean to selectively sum a scale?

**Response:** Thank you for pointing out this typo. We have removed the word “selectively”.

**Comment 3:** The presentation of results is still confusing. It appears that you proposed a scale and called it SES-RP. You present no empirical or theoretical justification for it being a single scale, nor do you point to it being an extant scale in the existing literature. You then conduct a CFA of these items and group them into five subscales, but do not present the CFA results that led you to that decision. Finally, based on correlation analysis that doesn't appear to treat the scales as latent constructs, you group the five scales into two scales based on what appears to be your interpretation of what makes sense along with the correlation analysis. I get the sense that you are using CFA in an exploratory rather than a confirmatory manner, but there is insufficient documentation. Why did some items have high factor loadings (presumably) when they were part of one subscale but had a statistically insignificant factor loading when they were part of another subscale? Finally, the naming of a subscale as "parent engagement" when all the scales, collectively, are referred to as parent engagement scales is confusing.

**Response:** Thank you for raising this issue. We have changed the layout of the manuscript. We agree, calling the school engagement items a scale (i.e. SES-RP) was incorrect. We now refer to these items as “school engagement items”. These items were used for the development of two scales namely: School Internal Engagement Scale-Refugee Parent (SIES-RP) and School Community Engagement Scale-Refugee Parent (SCES-RP) – see text in the results below. All references to the SES-RP have been removed. Since SES-RP is not a scale, CFA results are not presented. Later in the paper, we describe the items and CFA results for SIES-RP and SCES-RP scales respectively.

*“The CFA identified five distinct dimensions. These were communication between home and school, practical assistance provided by the school, parent participation in school activities, school as facilitating social capital, and school as enabling acculturation. The dimensions of communication, assistance, parent participation and social capital correlated significantly with each other (r =0.25 to 0.96, p<0.05). Conversely, acculturation significantly correlated with the social capital dimension (r = 0.68, p<0.01) but not with the communication or assistance dimensions (p > 0.05) (see Supplementary File S3). For this reason, the scale was divided into two theoretically and statistically distinct scales: (i) refugee parent engagement pertaining to communication, assistance and participation within the internal school system - named the School Internal Engagement Scale-Refugee Parent (SIES-RP) (16 items); and (ii) broader refugee parent engagement within the school community, recognising school as facilitating social capital and acculturation - named the School Community Engagement Scale-Refugee Parent (SCES-RP) (7 items). A CFA was performed on each separate scale. Final CFA model fitness indices are presented in Table 2. Eleven of the original 16 items were retained for the SIES-RP, whilst all 7 items of the SCES-RP were retained, (see supporting information files S4a and S4b, respectively).”*

**Comment 4:** A Cronbach's alpha of 0.67 for an 11-item scale is not typically acceptable, as the alpha is a function of both average inter-item correlation and the number of items—11 items is a large scale. This is also reflected in Supplemental Table 5, which shows that some of the items have low correlations with most of the other items and in Table 3's goodness of fit measures. It would be helpful to see estimates Cronbach's alpha with each item deleted one at a time to affirm that all items belong in the scale or to have a theoretical rationale for why these items should remain in the scale.
**Response:** Thank you for your suggestion. We examined the item deletion method to improve Cronbach's alpha for the 11-item SIES-RP. The item deletion process showed only marginal improvement of Cronbach's alpha from 0.67 to 0.68 (Cronbach's alpha values ranged from 0.64 to 0.68 using the item deletion technique). We have acknowledged this significant limitation in the discussion as per below:

*“The reliability of the SIES-RP and CBRS was below the acceptable threshold. Future waves of data will be useful to explore this further.”*

**Comment 5:** In short, the development of the scales still needs more empirical and theoretical justification. I cannot tell if the methods used in this version of the paper are sufficient because not all results were presented.

**Response:** We hope that the changes outlined above, in particular removing reference to the SES-RP, has addressed this concern. We have included all the relevant results in the revised manuscript.

**Comment 6:** Finally, I think the authors might have misunderstood the reviewers' request to take into account the clustering of students in schools. We were not asking for school fixed effects in the models (dummy variables for the schools), although the authors' justification that sample size did not allow for that doesn't really make sense (it is four variables). Rather, we were asking that the authors adjust the standard errors of the regression coefficients, which are underestimated due to the fact that the observations within schools are not independent (e.g., families in the same school work with the same cultural broker).  The authors have two choices to address this. They can include school fixed effects (one indicator for four of the five schools, with the fifth one serving as the referent group), or they can adjust the standard errors using the complex samples general linear model option in SPSS (or another software). This probably won't change the results substantively, but since all reviewers requested it, I recommend the authors do it.

**Response:** Thank you for your suggestions about adjusting for school effect. We have chosen the first option. In the revised analysis for LASSO regression we have created four indicators for five schools considering 5^th^ as reference group. We describe this in the Predictive Analyses section of the Methods as per below:

*“To adjust for school effects, school was entered as predictor variable. Based on five sampled schools A, B, C, D and E (designated letters for confidentiality); four indicators were created considering the 5th school (school E) as the reference group.”*

**Comment 7:** In addition, the paper uses terms loosely and vaguely, causing the reader to have to work too hard. Here are some examples in the beginning of the paper. Such examples continue throughout even though I do not note them. The authors switch language between involvement and engagement (note title and first sentence of discussion) without explicitly defining either one. Please choose a single term, define it, and stick to it.

**Response:** We have chosen the term “engagement”. I provide a definition of this in the opening paragraph and then stick to this phrasing throughout. Where appropriate, all references to “parent involvement” in the paper, have been consistently replaced with the “parent engagement”.

**Comment 8:** Line 76: take out the word "socio-demographic" and simply refer to “parent characteristics." Psychological distress is not a sociodemographic characteristic.

**Response:** “Socio-demographic" term has been replaced with "parent characteristics”, as per request. The phrasing has also been updated in the abstract.

**Comment 9:** Lines 81-82: Low variance explained does not mean a set of variables isn't important. I recommend taking out this statement.

**Response:** We have removed this statement.

**Comment 10:** Lines 87-92: The stated conclusions are not based in the findings of this paper. You provide no evidence of effectiveness of cultural brokers in this study.

**Response:** We have rephrased this to read: “*At the school level, results suggest that cultural brokers may be effective in targeting newly arrived families.”*

**Comment 11:** Lines 95-99: You say the study was done because schools integrate refugee families into communities, but there is nothing in your study about community involvement. You explain the connection well in the intro, but this edited-down version doesn't really make sense. Isn't the purpose to develop appropriate measures of school involvement for program development and evaluation?

**Response:** This section of the research summary has been amended as below:

*“Current measures of parent school engagement do not consider the unique context of refugee families. This study was undertaken to develop appropriate measures of refugee parents’ engagement with school. The intent is that the measures may support the development and evaluation of refugee programs in school.”*

**Comment 13:** Line 109: Casual or causal?

**Response:** It is ‘Causal’. We have corrected this.

**Comment 14:** Lines 151-153: Sentence doesn't make sense. What is a refugee field? Current wellbeing is affected by past trauma. Not sure what it means to say it includes past trauma.

**Response:** This sentence has been amended to: “Refugee parents’ wellbeing is affected by past trauma and displacement”.

**Comment 15:** Lines 158-159. You say: "Current measures may not capture this." What is "this"? Unclear.

**Response:** This sentence has been amended to: “*Current measures of parent school engagement may not capture these specific activities attuned to refugee families”.*

**Comment 16:** Line 169: What do you mean by "capture this relationship?" Do you mean measure the quality of this relationship? The content of interactions? Unclear.

**Response:** This sentence has been amended to: *“…no measure exists to capture the quality of this relationship between the cultural broker and parents.”*

**Comment 17:** Line 170: "The development of such measures is important." What measures? Throughout intro need to be very explicit about the constructs you are talking about, keeping the focus narrowly on the constructs you are proposing to measure. There is a tendency in this paper to keep broadening the scope, which ironically weakens the paper by diffusing the focus.

**Response**. This sentence has been amended to: “*The development of tools to measure refugee parents’ engagement with school is important.”* Attention has also been paid to the clarity of the general wording of the paper.

**Comment 18:** Line 174-175: "Context-specific measures also carry important implications for advancing efforts to improve refugee parents' engagement…" Unclear. What contexts are you talking about?

**Response:** The phrase “context-specific” has been removed. The sentences now read as: “*Targeted measures of impact that capture the distinct ways in which refugee parents might engage with schools are needed to monitor the success of these initiatives. Targeted measures also carry important implications for advancing efforts to improve refugee parents’ engagement in their children’s schooling in a tailored way that is most valuable to the family.”*

**Comment 19:** Line 181-183. Now you are talking about "traditional sense." Please, just use the names of the constructs this refers to, as you've never defined traditional constructs. Similarly, why call the new constructs "extras?" That minimizes their core importance.

**Response:** Traditional definitions are presented in paragraphs 2-3 of the introduction. This sentence has been rephrased to: “*To this end, the primary aim of the present study was to develop and validate self-report tools to measure refugee parents’ engagement with school. We draw on Epstein’s framework, and it extend it to include specific elements of engagement that foster families’ social and cultural capital [31, 32].”*

**Comment 20:** Line 191: I suggest not using the word profiling. For U.S. audiences that has very negative connotations and is never something to be recommended.

**Response:** Thank you. We have changed this word to “identifying”.

**Comment 21:** Line 251: What are generic school engagement scales? You haven't defined these prior to now.

**Response:** The word “generic” has been removed.

**Comment 23:** Lines 234-236: This sample size estimation is completely out of context. Statistical power must be estimated in the context of an intended analysis, and you haven't proposed one yet. What sort of coefficients are you referring to for what statistical model? This should be presented later in the data and analysis sections of the methods.

**Response:** We have recalculated our sample size within the context of the study. We have presented this in the Predictive Analyses section of the methods, as per below.

*“Assuming that the maximum standard deviation would not exceed 15 for the largest sub-scale total score for the project, and applying a 95% confidence interval along with a 3% margin of error, a sample size of 96 was deemed sufficient to reject the null hypothesis at the 0.05 probability level.”*

**Comment 24:** Lines 258-59: Don't need to state that qualitative data was transcribed or the software you used to analyze it. Not relevant or informative for this study. Better to cite the study so reader can find full details.

**Response:** This section has been edited down, and this sentence removed.

**Comment 25:** Line 264: Need a little more information on the qualitative pilot.

**Response:** A separate subheading has been given to “Item Development and Pilot”. This includes more information on the pilot as below:

*“The items were piloted with several parents enrolled in the study, according to the interview protocol. A few items were dropped due to ambiguity, where the interviewee had to explain the item further, or where the parents interpreted the items in different ways during administration. Based on this feedback and a roundtable discussion, consensus was reached on a final master list of items that was translated into Arabic to form a provisional set of school engagement items, a provisional set of items pertaining to the cultural broker-parent relationship, and a provisional set of items pertaining to parent sense of school belonging, as detailed below.”*

**Reviewer #3:**

The authors have addressed my concerns.
