# Supplementary material for: Psychological distress, resettlement stress, and lower school engagement among Arabic-speaking refugee parents in Sydney, Australia: A cross-sectional cohort study
Source: PLoS Med. 2021 Jul 12;18(7):e1003512. doi: 10.1371/journal.pmed.1003512 (PMC8312975; doi:10.1371/journal.pmed.1003512)
Supplement: Attachment — Submitted filename: 3rdResponsetoReviewers_final.docx [file pmed.1003512.s006.docx]

**Requests from the Editors**

**Comment 1:**

In order to comply with PLOS' data policy, we will need to ask that you provide a non-author contact for readers interested in inquiring about access to study data.

**Response:** We suggest:

The Human Ethics Coordinator

The University of New South Wales

Tel: 02 9385 6222

Email: humanethics@unsw.edu.au

Please let us know if you require this information to be provided in a certain format somewhere.

**Comment 2:** Please remove "are associated with" from your title so that it conforms to PLOS Medicine's style (i.e., non-declarative titles). Please mention the study setting in the title, e.g., "Psychological distress, resettlement stress and lower school engagement among Arabic-speaking refugee parents in Sydney, Australia: a cross-sectional study".

**Response:** Amended.

**Comment 3:** Please remove the information on funding and competing interests from the title page. In the event of publication, this information will appear in the article metadata via entries in the submission form.

**Response:** Removed.

**Comment 4:** Please adapt the "Methods and findings" subsection of your abstract to briefly describe the pilot phase of the study also, quoting quantitative details of the findings.

**Response:** The following information has been added to this subsection:

“Confirmatory factor analyses **were run on provisional item sets identified from a literature review and separate qualitative study. The findings** informed…”

**Comment 5:** Please include study dates in the abstract; and add a few words to make it clear what the "Kessler10" is. Please also quote aggregate demographic details for study participants in the abstract.

**Response:** This information has been added as below:

**“**Methods and Findings: The report utilises **2016** baseline data of a cohort study……. **Most participants were born in Iraq (81%) or Syria (11%) and only 25% spoke English well to very well** …

**… “**Parents’ psychological distress (as measured by the Kessler10 **self-report**)”.

**Comment 6:**

Please restructure the end of the "Methods and findings" subsection of your abstract so that the final sentence begins "Study limitations include ..." or similar.

**Response:** Amended as per below:

Study limitations include the cross-sectional design and the modest amount of variance (8-22%) accounted for by the regression models.

**Comment 7:** Please avoid claims of "the first" and the like, e.g., at line 83. Where needed, please add "to our knowledge".

**Response:** This sentence has been changed from:

*“The study presents the first refugee-specific measures of parental school engagement.”*

… to…

*“The study offers preliminary refugee-specific measures of parental school engagement.”*

We have also removed these sentences from the discussion:

- *“It is the first ever quantitative consideration of refugee parents’ school engagement, founded in stakeholder priorities.”*
- *“This is the first study to quantifiably identify poor parent mental health as a risk factor for lower school engagement.”*
- *“It is anticipated that the developed tools may be a cornerstone in advancing research into initiatives encouraging the meaningful engagement of refugee parents with their children’s school.”*

**Comment 8:** Please trim the "Conclusions" subsection of you abstract to no more than 3-4 sentences, and we ask you to focus the text more on the conclusions of the study and their immediate implications.

**Response:** We have addressed this by removing the last line of the abstract, as per below:

“The study offers refugee-specific measures of parental school engagement. It is expected they will provide a resource for evaluating efforts to support the integration of refugee families into schools. The findings support the need for initiatives that identify and support parents with school-attending children from refugee backgrounds who are experiencing psychological distress or resettlement stressors. At the school level, the findings suggest that cultural brokers may be effective in targeting newly arrived families. ~~At the federal level, policies that minimise the psychosocial challenges encountered by refugee families could help to improve parents’ capacity to engage with schools at the anticipated benefit to their children’s wellbeing.”~~

**Comment 9:** Please reformat the "author summary" so that each of the three subsections consists of 3-4 bulleted points, which should generally comprise a single sentence each.

**Response:** This has been amended as per below:

***“Why Was This Study Done?***

- *Current measures of parent school engagement do not consider the unique experiences of families from refugee backgrounds.*
- *This study was undertaken to develop appropriate measures of refugee parents’ engagement with school.*
- *It is expected that specific measures to ascertain refugee parents’ engagement will help support the development and evaluation of school-based refugee programs.*

***What Did the Researchers Do and Find?***

- *Using data from a cohort study of 233 Arabic-speaking refugee families, the study developed and validated four self-reports tools to assess parent school engagement and their sense of belonging.*
- *The study also explored the quality of the relationship between parents and the bilingual officer employed by schools to act as a cultural broker.*
- *Our findings suggest that parents’ who were experiencing psychological distress or post-migration living difficulties were less engaged with their child’s school.*

***What Do These Findings Mean?***

- *These new refugee-specific measures of parent engagement are expected to assist in evaluating programs aimed at supporting the integration of refugee parents and their children into schools.*
- *The cross-sectional design precludes causal interpretations.*
- *Nonetheless, the findings highlight a need to consider the identification and capacity-building of parents who are experiencing psychological distress or resettlement stressors.”*

**Comment 10:** Please add a heading to the Introduction.

**Response:** Amended

**Comment 11:** We ask you to restructure the Discussion section. The first paragraph should provide a summary of the paper's main findings, and there should be a separate discrete paragraph discussing study limitations.

**Response:** We have restructured the discussion as above. The first paragraph speaks to the main findings regarding the psychometrics of the developed measures. The second paragraph speaks to the sociodemographic findings. A discussion ensues, followed by two discrete paragraphs on limitations, then future research and implications. To keep this response manageable, rather than include the entire restructured discussion below, please see pp. 24-29 of the clean manuscript.

**Comment 12:** Please avoid "a couple of" at line 536.

**Response:** This phrase has been amended to “some”.

**Comment 13:** Please ensure that all p values are quoted exactly, or for smaller values as p<0.001.

**Response:** Amended in text.

**Comment 14:** Where appropriate, please substitute "sex" for "gender" throughout the paper.

**Response:** Amended

**Comment 15:** Please remove footnotes from your text: these can be integrated into the main text at suitable points. Acknowledgements to reviewers can appear in the acknowledgments section.

**Response:** Amended

**Comment 16:** Please remove all reference call-outs from subheadings, e.g., at line 272.

**Response:** Amended.

**Comment 17:** Throughout the text, please remove spaces from the reference call-outs (e.g., ... refugee students [5,6].").

**Response:**  Amended

**Comment 18:** Please revisit your reference list to ensure that all citations contain full access information and comply with journal format. We note that reference 76, for example, appears to lack full access details.

**Response:** We have revisited the reference list to ensure that the citations contain full access information.

**Comment 19:** Please reformat your supplementary material so that the study protocol and STROBE checklist are both separate supplementary documents, individually referred to in the Methods section (e.g., "See S1_STROBE_Checklist").

**Response:** The supplementary material has been amended as per guidelines stipulated below.

**Comments from Reviewers:**

***** Reviewer #1:**

The authors have addressed my concerns and I now recommend publication.

**Peter Flom**

***** Reviewer #2:**

The authors made some of the requested changes. I thank them for that.

I have some final suggestions.

**Comment 20:** There seems to be a suggestion that the new measures could be used to identify individual parents at risk of low engagement. The authors need to be careful about making that claim because developing tools for monitoring is very different from developing screening tools. Be careful to frame the purpose of the multivariate analysis as identifying risk factors (vs. parents at risk) for low engagement.

**Response:** This is an important distinction. We have read back through the paper. The revisions, particularly to the abstract and the discussion, and the improved clarity of wording we hope has now removed any ambiguity in relation to this.

**Comment 21:** p. 12. The description of the measure development is still confusing. This is primarily an organizational suggestion, but I think an important one. You are presenting the analytic strategy in the measures section. In measures section, I would have expected to see a statement describing the theoretical constructs you are testing with CFA and to be given examples of items that you believe measure each construct. This is missing from the methods section entirely. Then the CFA description should go in the analysis section, which you don't have. Consider adding a heading for Data Analysis (like you have for Measures) and putting all subsections starting with Measurement Development under that heading.

**Response:** We had adapted the structure accordingly. Example items can be seen under the headings in the methods of, “Refugee Parent School Engagement Items”, “Cultural Broker-Parent Relationship Items” of, “Refugee Parents’ Sense of School Belonging Items’. The full starting list of items is also now provided as a supplementary file.

**Comment 22:** p. 16 - 18. All of the information in the section Item Development and Pilot should be put in the Measures section. I thank the authors for adding this important information. This is the information I was expecting to see on page 12. I would move it into the Methods section, since you are describing the methods of your paper, not the results.

**Response:** We have moved this to the methods section, and added to the introuductory sentence to signpost readers:

*“Participants completed an extensive demographic survey followed by a provisional set of items for the school engagement measures, and then lastly, Arabic versions of the established measures below.”*

**Comment 23:** Line 412 - you hypothesized 3 domains but said CFA identified 5. Need more context here on what happened. How did you get to five and how did you name them what you named them? And then you go back to four scales. It is impossible to follow your decision-making here. I've stated this same limitation multiple times now. It is starting to feel futile.

**Response:** By separating the initial items into the methods section, and the CFA findings in the results section, we hope that this has assisted in explicating this. The understandable confusion is likely due to mixing up within and between the proposed measures. To aid comprehension of this, we have developed a flow diagram of the process development (see Figure 2), delineating each step for each measure. Furthermore, we have added the following description in the “Refugee Parents School Engagement” section in the results, as per below:

*“The 23-item measure followed five themes identified in the preliminary qualitative study (to be published elsewhere). The themes were (i) ease of communication between the school and parent (five items, e.g. “It would be difficult for me to find a way to discuss an issue with school staff”) ;(ii) practical assistance provided by the school such as accessing services or provision of food or uniforms (five items, e.g., “School staff direct me to services I need or that might be helpful to access”); (iii) degree of parent participation in school activities (six items, e.g. “I regularly attend school events”); and the way in which school facilitated (iv) social capital (four items, e.g. “Through the school, I have met and made friends”) or (v) acculturation (three items, e.g. “This school helps me feel like I belong in Australia”).*

**Comment:** Which leads me to my final point. I hope that going forward in their careers, the authors respond less superficially to reviewer comments than they did in the case of his manuscript. It is frustrating to keep saying the same changes are needed over and over. I am committed to this topic and the paper had potential, so I stuck it out. The authors would be better served and get their work published more quickly if they responded more fully to reviewer comments. I am not saying that the authors need to change their paper as the reviewer wishes -- a full response could include an explanation why the reviewer is incorrect in their assessment.

**Response:** Thank you for your patient input. We are grateful for the generous amount of time you have clearly spent on the paper, and we appreciate how much your constructive, tangible feedback has improved the paper. We acknowledge that we may not have understood and hence not responded fully to some of the comments. We hope that the current version has addressed all the comments fully.

----

**Production Issues**

FIGURES and GRAPHICS:

Please ensure that all main figures are referenced in the manuscript as Fig 1, Fig 2, etc. (including capitalization), rather than Figure 1, Figure 2, etc. Please note, however, that the file names themselves must not include the space, i.e. Fig1.tif, Fig2.tif. Please ensure that all main figure files are clearly labeled as such in the File Inventory.

**Response:** Amended.

METADATA:

**Comment:**

1. Please provide a detailed Financial Disclosure statement. This is published with the article, therefore should be completed in full sentences and contain the exact wording you wish to be published.

2. Please clarify all sources of funding (financial or material support) for your study. List the grants (with grant number) or organizations (with url) that supported your study, including funding received from your institution.

3. State the initials, alongside each funding source, of each author to receive each grant.

4. State what role the funders took in the study. If the funders had no role in your study, please state: “The funders had no role in study design, data collection and analysis, decision to publish, or preparation of the manuscript.”

5. If any authors received a salary from any of your funders, please state which authors and which funders.

If you did not receive any funding for this study, please state: “The authors received no specific funding for this work.”

**Response:** We have added a financial disclosure statement as follows:

“This research was part funded by the National Health and Medical Research Council of Australia (NHMRC) program grant (APP1073041) awarded to DS (CI) and SR (AI). The NHMRC had no role in study design, data collection and analysis, decision to publish, or preparation of the manuscript.”

**Comment:**

In accordance with PLOS' data policy, please ensure that your Data Availability Statement in the submission form clearly identifies how readers can access your data. Note that it will be typeset as it is written so please ensure it is in complete sentences and appears as you would like it to in the published version. For details on our data policy, please see <http://journals.plos.org/plosmedicine/s/data-availability>

**Response:** The data that support the findings of this study are available upon request from the study authors, with the permission from the Department of Education NSW.

**ARTICLE FILE:**

- Please indicate the corresponding author on the title page with an asterisk (*) and include the corresponding author's email address. Only an asterisk and the email address itself are required.
- Please remove author degree titles from the byline of your manuscript file.
- Please ensure that all authors have at least one affiliation listed on the title page and that all listed affiliations are attributed to at least one author. Affiliations should be listed in ascending numerical order in the affiliation list and should match what is provided in the Authors Information in the submission form.
- Affiliations should include an institution, a city, and a country (no postcode). US affiliations need the state as well, written out in full (with no abbreviations).
- Please ensure that all author names are spelled correctly in the manuscript file and the submission form, including the use of a middle name/initials, and any special characters (accents, umlauts, etc.). Please place first/given names before last/family names. We ask that you ensure they match verbatim. Please be aware that correct spelling and formatting of author names in the article file is vital for accurate indexing in PubMed.
- Please ensure that your Ethics Statement is available in the Methods section of your manuscript in its entirety, under the subheading "Ethics Statement".
- PLOS does not publish page footnotes. Please remove the footnotes and incorporate them into your text.

**Response:** All article file requests have been attended to.

**ACKNOWLEDGMENT**S:

1. According to PLOS policy, we ask that you please remove any mention of the reviewers or editors from the Acknowledgments section of your manuscript.

**Response:** Amended.

**REFERENCES:**

**Comment:** Please make sure all references are cited in ascending numerical order in the text.

**Response:** This has been adhered to.

**SUPPORTING INFORMATION:**

**Comment:**

1. Please upload your Supporting Information items individually, rather than in a single file, as you refer to them individually in the text. If you would like to retain the single file please rename internal items (e.g. tables) as A, B, C etc. Please also provide a legend for each under the heading Supporting Information. Supporting Information files should be less than 10 MB each. Please edit any citation of internal items within your Supporting Information files to using letters rather than numbers, e.g. “Table A in S1 Text”. Please ensure these changes are made throughout the manuscript and within the Supporting Information files themselves.

2. Please ensure that all Supporting Information files use the following format verbatim throughout the manuscript (including legends and all in-text citations): S1 Fig, S1 Table, S1 Text, S1 Data, etc. Each file type should start at 1 and increase numerically. Please note, however, that the file names themselves must use an underscore rather than a space, i.e. S1_Fig.tif, S1_Table.xlsx, S1_Text.doc, S1_Data.xlsx, S1_PRISMA_Checklist.docx, etc.

3. Please ensure that each supporting information file has a legend at the end of the manuscript file, after the Reference list.

4. Please note that your submission cannot contain any references to generic "supporting information" files and instead must refer to specific files using our naming convention (S1 Fig, S1 Table, S1 Text, S1 Data, etc.)

**Response:** Amended accordingly. Supporting files are uploaded as one document, with tables now named alphabetically rather than numerically.
